# Interstitial pneumonia with autoimmune features show better survival and less exacerbations compared to idiopathic pulmonary fibrosis

**DOI:** 10.1186/s12890-019-0868-9

**Published:** 2019-07-04

**Authors:** Jeong Uk Lim, Bo Mi Gil, Hye Seon Kang, Jongyeol Oh, Yong Hyun Kim, Soon Seog Kwon

**Affiliations:** 1 Division of Pulmonary and Critical Care Medicine, Department of Medicine, The Armed Forces Capital Hospital, Seongnam, South Korea; 2 Department of Radiology, Bucheon St Mary’s Hospital, College of Medicine, The Catholic University of Korea, Bucheon, South Korea; 3 Division of Pulmonary, Allergy and Critical Care Medicine, Department of Internal Medicine, Bucheon St. Mary’s Hospital, College of Medicine, The Catholic University of Korea, 327, Sosa-ro, Bucheon-si, Gyeonggi-do 14647 Republic of Korea

**Keywords:** Interstitial pneumonia with autoimmune features, Interstitial lung disease, Connective tissue disease, Idiopathic pulmonary fibrosis

## Abstract

**Background:**

Patients with interstitial lung disease (ILD) who show features related to autoimmunity without meeting criteria for a defined connective tissue disease are categorized as interstitial pneumonia with autoimmune features (IPAF). The present study compared clinical characteristics and clinical outcomes of patients with IPAF to patients with connective tissue disease related-interstitial lung disease (CTD-ILD) and patients with idiopathic pulmonary fibrosis (IPF).

**Methods:**

ILD patients who were consecutively enrolled in a single institution ILD cohort between 2008 and 2015 were evaluated for the study. Clinical data had been prospectively collected, while radiologic imaging and pathologic findings were re-reviewed for the present study.

**Results:**

Out of 305 patients with ILD, 54 (17.7%) patients met the classification of IPAF, 175 (57.4%) patients had IPF, and 76 (24.9%) patients were diagnosed with CTD-ILD. Compared to IPF, incidences of acute exacerbations in 1,3 and 5 years were significantly less in the IPAF group (*p* = 0.022, *p* = 0.026 and *p* = 0.007, respectively). From multivariate analysis for mortality, age (*p* = 0.034, HR 1.022, 95% CI: 1.002–1.044), FVC (*p* < 0.001, HR 0.970, 95% CI: 0.955–0.984), ILD exacerbation (*p* = 0.001, HR 2.074, 95% CI: 1.366–3.148), and ILD type (*p* = 0.047, HR 0.436, 95% CI: 0.192–0.984 (IPAF vs IPF), respectively) showed significant association.

**Conclusions:**

Compared to the other ILD groups, IPAF showed distinct clinical characteristics. The IPAF group showed better survival and less episodes of exacerbation when compared to the IPF group.

## Background

Interstitial pneumonia with autoimmune features (IPAF) is a conceptual entity proposed to identify patients with interstitial pneumonia and features suggestive of connective tissue disease (CTD), but not meeting established classification criteria for CTD [1]. The traditional serologic and clinical features of connective tissue disease (CTD) were included in the proposed criteria of IPAF. The findings from high-resolution computed tomography (HRCT), histopathology and other diagnostic modalities such as pulmonary function tests and echocardiography which were consistent with CTD, were included in the morphologic domain of IPAF [2].

IPAF criteria were applied to patients previously diagnosed as undifferentiated-CTD interstitial lung disease (UCTD-ILD) [3]. According to previous publications, majority of IPAF patients were shown to be female and had no or little smoking history [4, 5]. Furthermore, the most frequent ILD pattern on HRCT was non-specific interstitial pneumonia (NSIP) [6, 7]. The clinical outcomes of IPAF have been compared to other ILD types. From the study by Oldham et al., the IPAF cohort showed worse survival than the CTD-ILD group, while showing slightly better survival than the idiopathic pulmonary fibrosis (IPF) group [3]. The study by Ahmad et al. found no significant difference between IPAF and IPF [7].

However, in order to recommend the classification of IPAF for wider use among clinicians, more clinical data needs to be accumulated. A series of studies on IPAF were published after the expert consensus in 2015, however, little is known about longitudinal clinical outcomes of IPAF, and the question whether IPAF has better survival than IPF remains to be answered. Furthermore, the acute exacerbation (AE) of ILD has been reported to be a significant negative prognostic factor associated with worse survival in CTD-ILD and IPF [8–10]. Nevertheless, it has not been evaluated for IPAF in previous studies.

In the present study, we compared clinical characteristics, survival and ILD exacerbation of IPAF patients to the CTD-ILD and IPF groups, from the ILD cohort of a single institution.

## Method

### Patient selection

ILD patients who were consecutively enrolled at the time of diagnosis between 2008 and 2015 were evaluated. Clinical data was collected prospectively in this ILD cohort. Our institution has a longitudinal ILD cohort and the clinical and laboratory data of patients in this cohort have been collected prospectively using an ILD-pre-specified protocol. Databases were collected on a regular basis and in real-time at the time of work up. For the present study, data has been retrospectively reviewed, while radiologic imaging and pathologic findings were re-reviewed by radiologists and pathologists.

According to the European Respiratory Society/American Thoracic Society research statement on IPAF, 54 patients were categorized into the IPAF group [1]. All CTD-ILD patients had been referred to rheumatologists before diagnosis. Clinical characteristics and overall survival of the IPAF patients were compared to those of CTD-ILD patients and IPF patients. For comparison of clinical characteristics, the IPF group was further categorized into seronegative and seropositive IPF subgroups.

### IPAF criteria

The diagnosis of IPAF was done following the European Respiratory Society/American Thoracic Society research statement [1]. The patients’ clinical data, laboratory, radiologic and histopathologic findings were evaluated to check whether they met the criteria of clinical, serologic and morphologic domains. After exclusion of other possible causes, such as malignancy or heart failure, initial HRCT images were checked for features of multi-compartment involvement such as unexplained pericardial effusion, and pleural effusion and thickening. Unexplained intrinsic airways disease was defined as never smokers with forced expiratory volume in one second/forced vital capacity (FEV1/FVC) ratio of < 70%. We defined it as such, as similar findings in ever-smokers would mean a possibility of concurrent chronic obstructive pulmonary disease [11]. Pulmonary vasculopathy was determined by the presence of pulmonary hypertension (PH) defined as a mean pulmonary artery pressure ≥ 25 mmHg via right heart catheterization (RHC) [1]. RHC is the gold standard diagnostic method for the diagnosis of PH, but we did not perform RHC routinely as this is an invasive procedure [12]. In the present study, findings coherent to high right ventricular load detected on trans-thoracic Doppler echocardiography [13] or FVC/diffusing capacity for carbon monoxide (DLco) > 1.6 and DLco < 60% were used as criteria for the definition of pulmonary hypertension [1, 3, 14]. In order to exclude the possibility of an overlap with CTD-ILD, all our potential IPAF patients were seen by rheumatologists.

### Time to first exacerbation

AE of non-IPF ILD groups and IPF group were defined using the revised definition of IPF proposed in 2016 [15]. There is, however, no existing official definition of AE-ILD in non-IPF ILD [16]. Time duration between enrolment to the cohort and first ILD exacerbation was estimated. An exacerbation of ILD was defined as a patient being admitted to the hospital due to acute aggravation of ILD occuring less than 1 month before admission [15].

### Seropositive IPF

Patients with IPF who presented with positive features of the serologic domain, but without any features of the clinical and morphologic domains of IPAF were classified as the seropositive IPF. Patients with IPF who did not have positive features of the serologic domain of IPAF were classified as seronegative IPF.

### Statistical analysis

For comparison of continuous variables between the groups, one-way analysis of variance (ANOVA) was performed, and chi-squared test was used to compare categorical variables. Overall survival (OS) was estimated from the time of enrolment in the cohort until death of any cause. Kaplan-Meier curve analysis and log-rank test were used to compare duration of survival between the groups. For survival analysis, cox regression hazard model was used to evaluate association between mortality and the clinical variables. The factors which were significant in the univariate analysis were entered into a multivariate Cox regression model to determine their independent effects. Statistical significance was established at a *P*-value of 0.05.

### Ethical statement

This retrospective study was approved by the Institutional Review Board of Bucheon St Mary’s Hospital. The need for informed consent was waived because the study was a retrospective review.

## Results

### IPAF characterization

Among 305 patients evaluated, 54 (17.7%) patients met the classification of IPAF. Table 1 shows detailed features within each domain of IPAF patients. A total of 17 (31.5%) patients showed features coherent with the criteria of the clinical domain. Arthralgia was the most common symptom (76.5%), followed by Raynaud’s phenomenon and unexplained digital oedema (both 16.7%). Among 54 IPAF patients, 49 (90.7%) patients met the criteria of the serologic domain. ANA abnormality was the most common finding (63.3%), followed by high rheumatoid factor levels and positive anti-cyclic citrullinated peptide (anti-CCP) (14 (28.6%) and 7 (14.3%) patients, respectively). A total of 44 (81.5%) patients met the morphologic domain criteria. Among the 39 patients with features of the morphologic domain, 34 (87.2%) patients showed NSIP patterns on HRCT findings, which was the most common ILD pattern in the IPAF group. Three (7.7%) patients presented with organizing pneumonia. Among 34 patients who underwent diagnostic biopsy, 12 patients showed histopathologic patterns consistent with features of the morphologic domain. In 5 patients, interstitial lymphoid aggregates with germinal centres were the most common finding (41.7%) among patients with histopathologic patterns related to IPAF, followed by pathologic findings consistent with organising pneumonia (4 patients, 33.3%). Findings defined as multi-compartment involvement were seen in 11 (20.4%) patients; unexplained pleural effusion or thickening in 5 (45.5%) patients, unexplained pericardial effusion or thickening in 3 (27.3%) patients, unexplained intrinsic airways diseases in 2 (18.2%) patients and unexplained pulmonary vasculopathy in 1 (9.1%) patient.

**Table 1:** Autoimmune features of 54 IPAF patients according to criteria and domains

Clinical domain (*n* = 17)	
Distal digital fissuring	1 (5.9)
Distal digital tip ulcerations	0 (0)
Inflammatory arthritis or polyarticular morning joint stiffness ≥60 min	13 (76.5)
Palmar telangiectasia	0 (0)
Raynaud’s phenomenon	3 (17.6)
Unexplained digital oedema	3 (17.6)
Unexplained fixed rash on the digital extensor surface	0 (0)
Serologic domain (*n* = 49)	
ANA ≥1:320 titer, diffuse, speckled or homogeneous patterns, ANA nucleolar pattern (any titer), or ANA centromere pattern (any titer)	31 (63.3)
Rheumatoid factor > 2x upper limit of normal	14 (28.6)
Anti-CCP	7 (14.3)
Anti-dsDNA	3 (6.1)
Anti-Ro (SS-A)	4 (8.2)
Anti-La (SS-B)	1 (2.0)
Anti-topoisomerase (Scl-70)	1 (2.0)
Anti-ribonucleoprotein	2 (4.1)
Anti-Smith	2 (4.1)
Anti-tRNA synthetase, Anti-Pm-Scl, Anti-MDA-5	0 (0)
Morphologic domain (*n* = 44)	
Suggestive radiology patterns by HRCT	39 (72.2)
Nonspecific interstitial pneumonia	34 (87.2)^a^
Organising pneumonia	3 (7.7)^a^
Nonspecific interstitial pneumonia with organising pneumonia overlap	2 (5.1)^a^
Lymphoid interstitial pneumonia	0 (0)^a^
Histopathologic pattern	12 (22.2)
Nonspecific interstitial pneumonia	0 (0)^a^
Organising pneumonia	4 (33.3)^a^
Nonspecific interstitial pneumonia with organising pneumonia overlap	1 (8.3)^a^
Interstitial lymphoid aggregates with germinal centres	5 (41.7)^a^
Diffuse lymphoplasmacytic infiltration	2 (16.7)^a^
Multi-compartment involvement (in addition to interstitial pneumonia)	11 (20.4)
Unexplained pleural effusion or thickening	5 (45.5)^a^
Unexplained pericardial effusion or thickening	3 (27.3)^a^
Unexplained intrinsic airways diseases	2 (18.2)^a^
Unexplained pulmonary vasculopathy	1 (9.1)^a^

Abbreviations: *HRCT* high-resolution computed tomography scan, *IPAF* interstitial pneumonia with autoimmune features

^a^Percentages show the proportions of positive findings among each subdomain in which they are included (suggestive radiology patterns by HRCT, histopathologic pattern, and multi-compartment involvement)

### Comparison of clinical characteristics between IPAF, CTD-ILD and seronegative/seropositive IPF

Clinical characteristics of the IPAF patients were compared to 76 CTD-ILD, 145 seronegative IPF and 30 seropositive IPF (Table 2) patients. The CTD-ILD group was comprised of 46 patients with rheumatoid arthritis (RA), 18 patients with systemic sclerosis, 6 patients with Sjogren’s syndrome, 5 patients with inflammatory muscle disease and 1 patient with systemic lupus erythematous (SLE).

**Table 2:** Comparison of clinical characteristics between IPAF, CTD-ILD and IPF

	IPAF (*n* = 54)	CTD-ILD (*n* = 76)	Seronegative IPF (*n* = 145)	Seropositive IPF (*n* = 30)	*P*-value
Sex (male) (*n*, %)	19 (35.2)	24 (31.6)	103 (71.0)	22 (73.3)	< 0.001
Mean age (SD)	67.9 ± 10.5	61.6 ± 13.5	71.6 ± 9.5	71.8 ± 8.3	< 0.001
Ever smoker (*n*, %)	15 (27.8)	23 (30.3)	95 (65.5)	20 (66.7)	< 0.001
Smoking pack years	7.0 ± 14.9	11.1 ± 20.4	24.6 ± 23.1	26.7 ± 32.1	< 0.001
ILD pattern from HRCT					< 0.001
UIP	14 (25.9)	35 (46.1)	145 (100)	30 (100)	
NSIP	34 (63.0)	17 (22.4)	0 (0)	0 (0)	
OP	3 (5.6)	5 (6.6)	0 (0)	0 (0)	
NSIP + OP	2 (3.7)	3 (3.9)	0 (0)	0 (0)	
LIP	0 (0)	2 (2.6)	0 (0)	0 (0)	
Emphysema from HRCT (*n*, %)	5 (9.3)	17 (22.4)	45 (31.0)	9 (30)	0.006
Lung biopsy at diagnosis^a^					< 0.001
None	20 (37.0)	33 (43.4)	86 (59.3)	14 (46.7)	
TBLB	13 (24.1)	11 (14.5)	38 (26.2)	12 (40)	
VATS	25 (46.3)	21 (27.6)	18 (12.4)	9 (30)	
FVC, L	2.4 ± 0.7	2.6 ± 0.8	2.5 ± 0.8	2.8 ± 0.9	0.063
FVC (% of predicted)	81.8 ± 17.0	86.2 ± 18.4	80.7 ± 19.1	83.8 ± 17.6	0.225
FEV1, L	1.9 ± 0.6	2.0 ± 0.6	2.0 ± 0.6	2.2 ± 0.7	0.127
FEV1/FVC	82.0 ± 7.7	79.1 ± 9.4	82.1 ± 8.9	79.9 ± 9.6	0.109
TLC, L	3.8 ± 1.2	4.1 ± 1.0	4.4 ± 1.4	4.5 ± 1.3	0.077
TLC (% of predicted)	87.8 ± 21.6	91.1 ± 18.7	91.6 ± 24.5	84.5 ± 19.0	0.434
VC,L	2.4 ± 0.7	2.6 ± 0.8	2.7 ± 0.8	2.8 ± 0.9	0.116
VC (% of predicted)	84.5 ± 17.7	87.4 ± 19.5	80.6 ± 19.2	82.0 ± 18.3	0.224
DLCO (absolute)	10.6 ± 4.4	10.6 ± 3.6	11.4 ± 6.0	9.7 ± 4.4	0.361
DLCO (% of predicted)	62.7 ± 21.0	62.3 ± 18.2	68.5 ± 24.3	57.9 ± 19.0	0.059

Abbreviations: *CTD-ILD* connective tissue disease-related interstitial lung disease, *DLCO* Diffusing capacity of the lungs for carbon monoxide, *FEV1* forced expiratory volume in 1 s, *FVC* forced vital capacity, *HRCT* high-resolution computed tomography scan, *ILD* interstitial lung disease, *IPAF* interstitial pneumonia with autoimmune features, *IPF* idiopathic pulmonary fibrosis, *LIP* lymphocytic interstitial pneumonia, *NSIP* nonspecific interstitial pneumonia, *OP* organizing pneumonia, *SD* standard deviation, *TBLB* transbronchial lung biopsy, *TLC* total lung capacity, *UIP* usual interstitial pneumonia, *VATS* video-assisted thoracoscopic surgery, *VC* vital capacity

^a^Some patients underwent multiple diagnostic procedures

The proportion of male (35.2%) patients was significantly lower in the IPAF group than in both the seronegative and seropositive IPF groups (*p* < 0.001, 71.0 and 73.3%, respectively), but similar to that of the CTD-ILD (31.2%) group. Mean age and the proportion of ever smokers was significantly lower in both the IPAF and CTD-ILD groups than in the seronegative and seropositive IPF (*p* < 0.001 and *p* < 0.001, respectively) groups.

There was a significant difference between the groups for ILD patterns observed on the initial HRCT (*p* < 0.001). In the IPF group, 100% of the patients presented with UIP patterns on HRCT and no radiological NSIP pattern was noted. Usual interstitial pneumonia (UIP) pattern on the initial HRCT was seen in 25.9% of the IPAF group and 46.1% of the CTD-ILD group. The NSIP pattern was the most frequent ILD pattern observed in the IPAF group (63%). While only 22.4% of the CTD-ILD group showed the NSIP pattern, which was the second most frequent pattern after UIP. Emphysematous change was seen in only 9.3% of the IPAF group, which is lower than in the CTD-ILD group, and the seropositive and seronegative IPF groups (*p* = 0.006).

In the IPAF group, 63% of the patients underwent diagnostic biopsy, which was the highest proportion among the four groups (*p* < 0.001). There was no difference in spirometric parameters between the four groups.

### ILD exacerbations

During the observation period, AE-ILD was seen in 25.9% of the IPAF group, 32.9% of the CTD-ILD and 35.4% of the IPF group (*p* < 0.001). The proportions of patients who experienced exacerbations were significantly different in 1, 3 and 5 years after enrolment in the cohort (*p* = 0.022, *p* = 0.026 and *p* = 0.007, respectively). When time to first exacerbation was compared between the groups, the IPAF group showed no significant difference compared to the IPF group, while the CTD-ILD group showed a significantly longer duration compared to the IPF group (*p* = 0.02). Mean value of time to first exacerbation was 29.5 ± 27.5 months in the IPAF group, 32.6 ± 29.7 months in the CTD-ILD group and 17.3 ± 21.4 months in the IPF group (Table 3).

**Table 3:** Comparison of clinical outcomes between IPAF, CTD-ILD and IPF

	IPAF (*n* = 54)	CTD-ILD (*n* = 76)	IPF (*n* = 175)	*P*-value*
Total deaths during observation	15 (27.8%)	16 (21.1%)	111 (63.4%)	< 0.001^ab^
Mean survival time (months)	73.3 ± 6.6	104.0 ± 6.7	52.0 ± 3.6	< 0.001^ab^
Time to first exacerbation (mean, months)	29.5 ± 27.5	32.6 ± 29.7	17.3 ± 21.4	0.02^a^
ILD exacerbations				
Whole observation period	14 (25.9%)	25 (32.9%)	62 (35.4%)	< 0.001^b^
5 yr	11 (21.1%)	19 (25.3%)	56 (33.5%)	0.007^a^
3 yr	9 (17.3%)	15 (20.0%)	47 (28.1%)	0.026^a^
1 yr	6 (11.5%)	9 (12.0%)	37 (22.0%)	0.022^a^

Abbreviations: *CTD-ILD* connective tissue disease-related interstitial lung disease, *ILD* interstitial lung disease, *IPAF* interstitial pneumonia with autoimmune features, *IPF* idiopathic pulmonary fibrosis

^*^Statistical difference between the three groups

^a^Significant statistical difference between CTD-ILD and IPF

^b^Significant statistical difference between IPAF and IPF

### Survival analysis

**Fig. 1:**
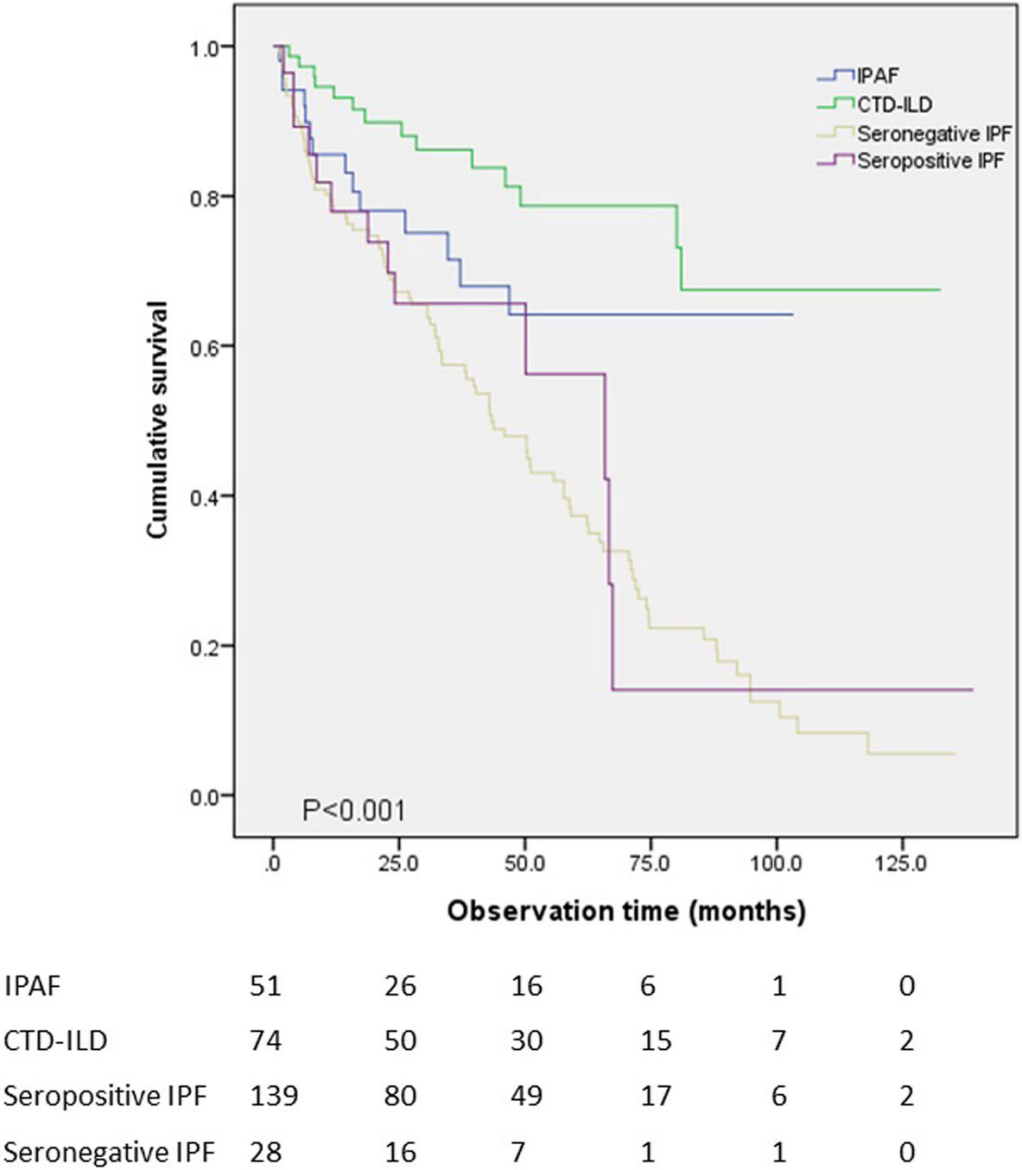
Overall survival was compared between the IPAF, CTD-ILD, seronegative IPF and seropositive IPF groups. Statistically significant difference was present between the four groups (*p* < 0.001)

**Fig. 2:**
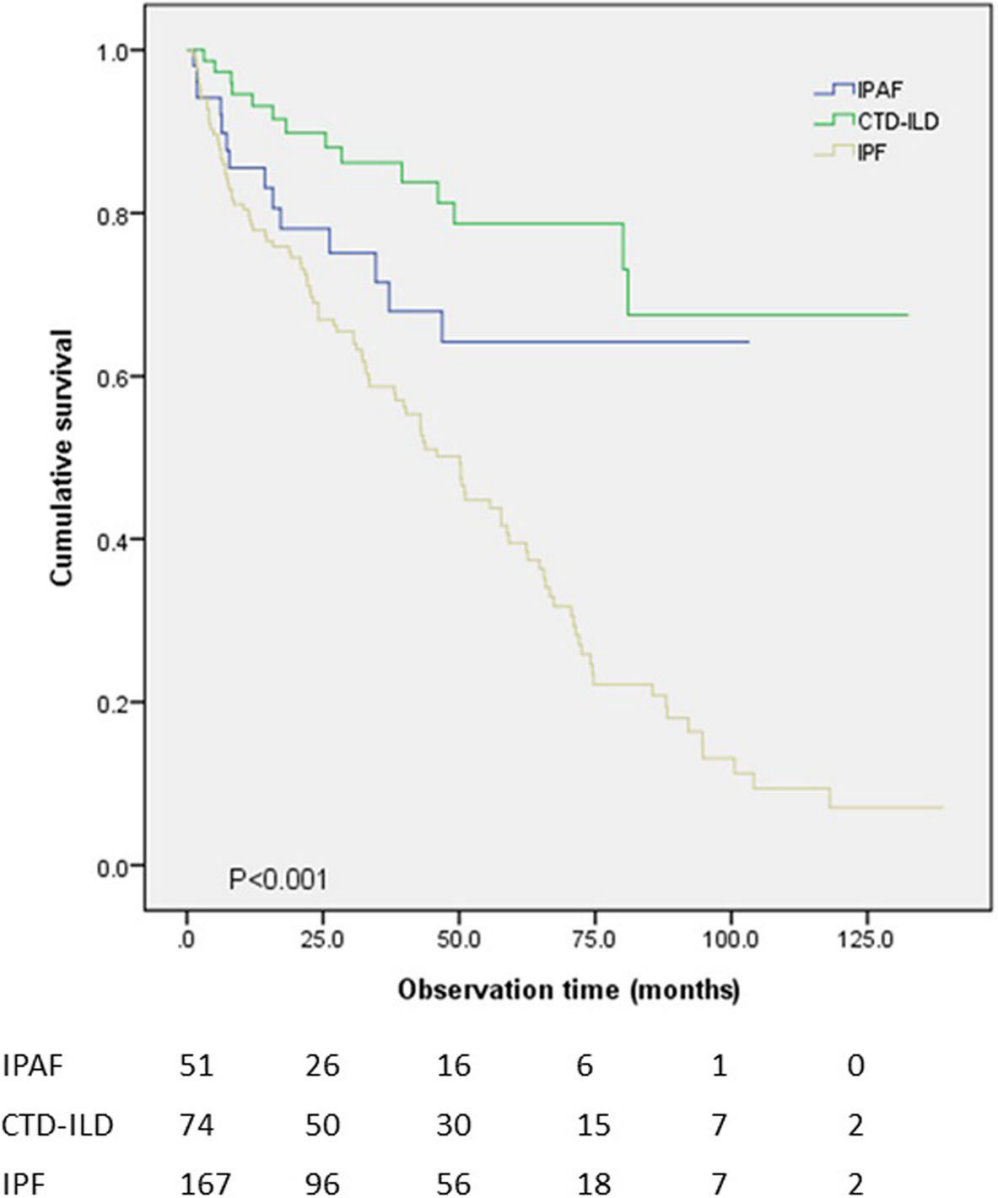
Kaplan-Meier analysis showed that the three groups showed significant difference in survival (*p* < 0.001). The IPF group was taken as a single group, regardless of seropositivity

**Fig. 3:**
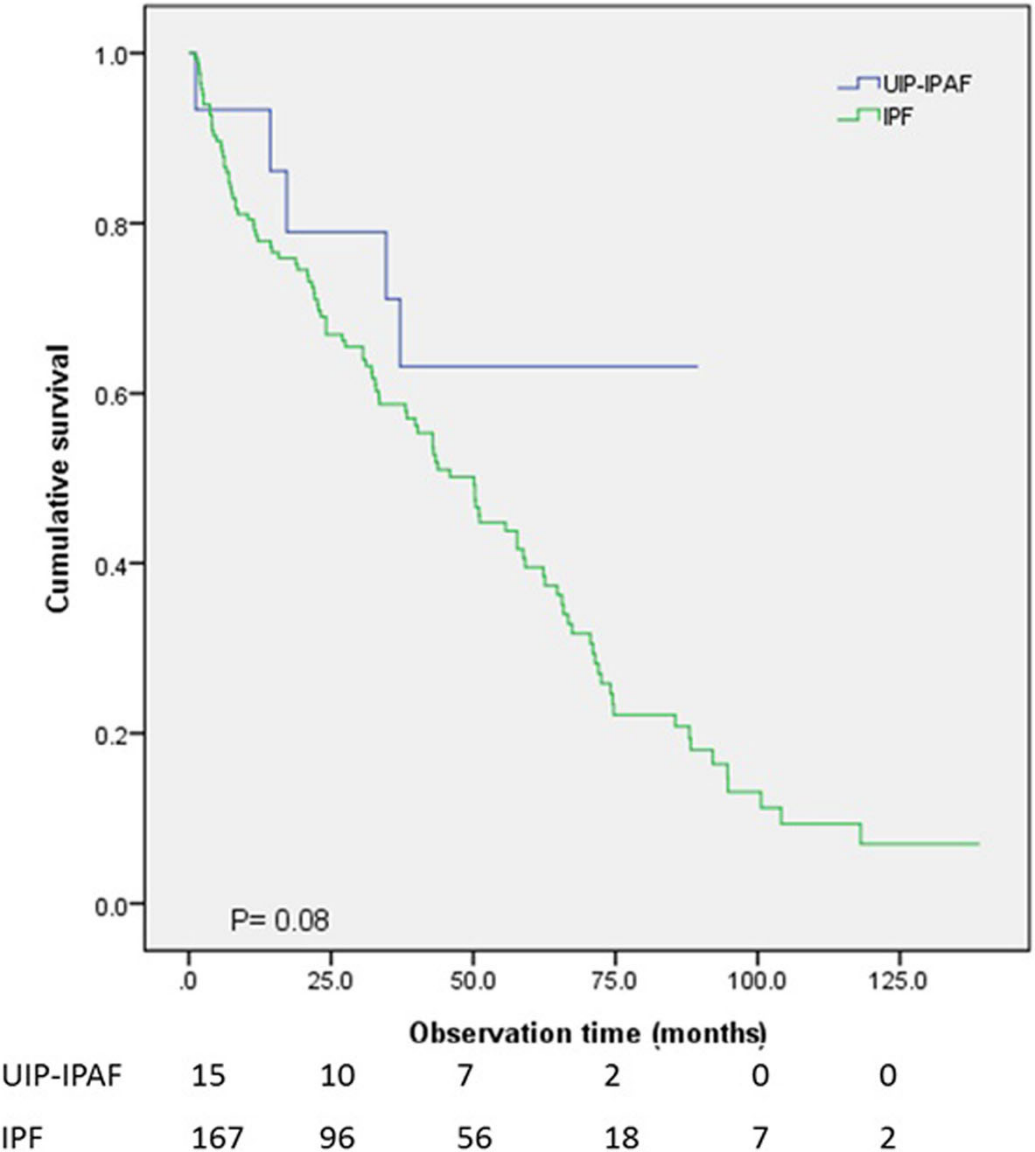
Survival was compared between the IPAF patients with UIP pattern (*n* = 15) and the IPF group (*n* = 175). No significant difference was present (*p* = 0.08)

OS was compared between the different combinations of ILD types. Mean survival time was 73.3 months in the IPAF group, 104.0 months in the CTD-ILD, 50.7 months in the seronegative IPF group, and 56.5 months in the seropositive IPF group. Difference on OS among the four groups was statistically significant (*p* < 0.001) (Fig. 1). When the seronegative and seropositive IPF groups were combined into a single group, a significant difference in OS of the three groups was seen as well (*p* < 0.001) (Table 3) (Fig. 2), with a mean survival time of 52.0 months in the IPF group. When the IPAF patients with UIP pattern (*n* = 15) were compared to the IPF group, OS was slightly better in the IPAF than in the IPF group (64.6 months vs 52.0 months, respectively), with no significant difference (*p* = 0.08) (Fig. 3).

For analysis of association with mortality, the following variables - age, gender, smoking history, autoimmune antibody positivity, first line treatment, emphysema from HRCT, FVC, DLCO, whether patients experienced exacerbation and ILD type, were entered in the univariate analysis. Age, gender, smoking history, autoimmune antibody positivity, FVC, DLCO, ILD exacerbation and ILD type were significant factors from the univariate analysis, and were entered in the multivariate analysis. Age, FVC, ILD exacerbation and ILD type were significant risk factors for mortality in the multivariate analysis (*p* = 0.034, HR 1.022, 95% CI: 1.002–1.044; *p* < 0.001, HR 0.970, 95% CI: 0.955–0.984; *p* = 0.001, HR 2.074, 95% CI: 1.366–3.148; *p* = 0.047, HR 0.436, 95% CI: 0.192–0.984 (IPAF vs IPF), respectively). From the multivariate analysis, the IPAF type was associated with significantly better survival when compared to the IPF (Table 4).

**Table 4:** Variables analyses for prediction of mortality in the study patients

	Univariate			Multivariate		
Characteristics	P	HR	95% CI	P	HR	95% CI
Age	< 0.001	1.035	1.018–1.052	0.034	1.022	1.002–1.044
Male/Female	0.011	1.563	1.106–2.210	0.945	0.976	0.493–1.934
Smoking history	0.032	1.463	1.034–2.071	0.110	1.758	0.879–3.513
Positive autoimmune Ab	< 0.001	0.498	0.347–0.714	0.433	0.804	0.467–1.386
First line treatment	0.549					
Systemic corticosteroid		1	–			
Antifibrotics		1.417	0.453–4.432			
Emphysema from HRCT	0.514	1.132	0.780–1.642			
FVC (%)	< 0.001	0.964	0.954–0.975	< 0.001	0.970	0.955–0.984
DLCO (%)	< 0.001	0.978	0.969–0.988	0.051	0.988	0.976–1.000
ILD exacerbation (yes/no)	0.003	1.654	1.180–2.317	0.001	2.074	1.366–3.148
ILD type	< 0.001			0.039		
IPF		1			1	
IPAF	0.008	0.470	0.269–0.821	0.047	0.436	0.192–0.990
CTD-ILD	< 0.001	0.235	0.135–0.410	0.026	0.401	0.179–0.898

Abbreviations: *Ab* antibody, *CTD-ILD* connective tissue disease-related interstitial lung disease, *DLCO* Diffusing capacity of the lungs for carbon monoxide, *FVC* forced vital capacity, *HRCT* high-resolution computed tomography scan, *ILD* interstitial lung disease, *IPAF* interstitial pneumonia with autoimmune features, *IPF* idiopathic pulmonary fibrosis

## Discussion

We applied the definition of IPAF in patients with ILD from the cohort at our institution, and further compared clinical characteristics and clinical outcomes of this cohort to patients with CTD-ILD and IPF from the same institution. Our study has its strength in comparing longitudinal clinical outcomes, including ILD exacerbation between the different ILD groups. In the present study, IPAF showed distinct clinical characteristics when compared to other ILD types. In addition, the IPAF group showed better survival and less exacerbation events when compared to the IPF group.

Of the three domains of the IPAF criteria, features of the serologic domain was most commonly observed (90.7%), thereafter, features of the morphologic domain (81.5%). This is different from the results of a previous study by Yoshimura et al. in which features of the morphologic domain was most commonly observed (96.9%), followed by the serologic domain [17], and consistent with the results of the study on IPAF by Ahmad et al. [7].

When baseline clinical characteristics were compared, the IPAF group in our study showed some similarities to previous studies. In our study, NSIP was the most frequent HRCT pattern, which was consistent with previous studies [7, 17]. Idiopathic NSIP is often associated with autoimmune features [6], and a high proportion of NSIP in the IPAF group suggests a possible correlation with autoimmune features.

Furthermore, the percentage of female patients was more than 50% for both the IPAF group and the CTD-ILD group. A female predominance in the IPAF group of our study is consistent with results of previous publications [5, 6]. However, Ahmad et al. demonstrated a slight male predominance in the IPAF group as well as the IPF group (female percentage 49%) [7]. Regarding smoking history, our study showed a lower proportion of ever smokers than previous studies. (27.8% vs 34.8 and 38.8%) [7, 18].

In addition to comparison of baseline clinical characteristics, our study focused on longitudinal clinical outcomes of IPAF: AE-ILD and mortality. First, the proportions of patients who experienced exacerbations in the IPAF group at different time periods were significantly lower when compared to the IPF group. Moreover, despite statistical insignificance, the time to first exacerbation in the IPAF group was longer than that of the IPF group. In IPF, AE-ILD is a known significant negative prognostic factor [8], and was found to be a significant factor associated with mortality in our multivariate analysis.

Secondly, our study demonstrated that the IPAF group showed significantly better survival than the IPF group. Ahmad et al. showed that there was no significant difference in OS between patients with IPAF and patients with IPF [7]. In addition, Oldham et al. showed that there was no statistically significant difference in OS between the IPAF and IPF cohort. On the other hand, both studies by Ahmad et al. and Oldham et al. showed that the survival of patients with IPAF was not better than that of patients with CTD-ILD [3, 7].

The finding that the IPAF group showed better survival compared to the IPF group should be considered in conjunction with the difference in ILD exacerbation incidence and proportion of UIP pattern observed on the initial HRCT. We first hypothesized that ILD exacerbation would be the major factor contributing to the difference of overall survival between the ILD groups, because AE-ILD is a strong negative prognostic factor in IPF [8]. Furthermore, compared to the IPF group, the proportion of patients who experienced AEs was significantly less in the IPAF group and this difference in proportion was repeatedly shown in year 1, 3 and 5. In addition, IPAF was independently associated with better survival compared to IPF, with other factors such as AE-ILD also adjusted in the analysis. However, the definition of AE in IPF was also applied to non-IPF groups in the present study [14], and this may have affected the analysis. Further studies, including more detailed evaluation of AEs in non-IPF ILD are needed to clarify other intrinsic factors associated with survival in IPAF.

We initially assumed that the proportion of UIP pattern observed on HRCT would influence the clinical outcome, because the UIP pattern has been reported to be associated with worse survival [19, 20]. In our study, survival comparison between the 15 IPAF patients with UIP pattern and the IPF group showed no significant difference (*p* = 0.08). The IPAF group tends to have a longer survival duration than the IPF group, however, the difference was not statistically significant. We believe that a future prospective study focusing on comparing these two specific subgroups may give a clearer comparative result.

We also attempted to compare the IPAF group to groups of other ILD types with different levels of autoimmune features. When the diagnostic criteria of IPAF were first recommended by ATS/ERS, they focused on ILD with autoimmunity, not meeting established criteria of CTD [1]. From our study, the IPAF group showed some clinical findings similar to CTD-ILD, such as sex, smoking history and time to first exacerbation. We believe that the similarities came from autoimmune tendencies shared by the two disease entities. Furthermore, IPAF patients should be placed under longitudinal surveillance for future occurrence of CTD [18, 21]. We think that patients who were categorized as IPAF at the time of initial diagnostic work up, should be re-evaluated on a regular basis for the possibility of definitive CTD-ILD. We also compared clinical characteristics of the IPAF group to the IPF group with seropositivity, considering the possibility that the latter group may have some autoimmune features. However, the seropositive IPF group showed no clinical similarities with the IPAF and the CTD-ILD groups in terms of sex, smoking history, radiologic findings and clinical outcomes. In addition, the seropositive IPF group did not show distinct clinical characteristics from the seronegative IPF group. Despite the serologic findings, we believe that the IPAF group and IPF group with autoimmune antibody share little similarity in clinical characteristics.

IPAF is a relatively recently defined disease category of ILD, and the applicability of the criteria has been repeatedly discussed [21–23]. From our study, arthralgia and Raynaud’s phenomenon were the two most common findings among the criteria of the clinical domain. This finding was similar to a previous study [7]. However, distal digital fissuring (“mechanic’s hands”), unexplained fixed rash on the digital extensor surfaces (Gottron’s sign), and distal digital tip ulceration were absent or less frequent signs in our study. We assume that some findings related to the clinical domain are more suggestive of CTD rather than fulfilling the diagnostic criteria for IPAF. As was suggested by Ahmad et al., these signs often lead to the diagnosis of CTD after referral to rheumatologists [7]. On the other hand, there were 10 patients from our ILD cohort who showed dry mouth or eye symptoms and also showed seropositivity or radiologic/histopathologic findings coherent to the morphologic domain of IPAF, but not fulfilling the diagnostic category of IPAF. Dry eye or dry mouth are clinical symptoms related to Sjogren’s disease [24]. Such symptoms are subjective, because they are non-visual findings and can vary depending on patients’ general condition. However, the symptoms of dry eye and dry mouth can be clinically relevant if other rheumatologic findings are present and should be checked for relevance to autoimmune features. We think that clinical findings such as dry eye or dry mouth could be carefully considered for inclusion into the clinical domain if concurrent autoimmune features are present.

The present study has some limitations. First, this is a single institution retrospective study, and the possibility of selection bias exists. However, the study patients were enrolled consecutively and many of the previous studies were also single institution-based studies [5, 7]. Secondly, categories other than CTD-ILD and IPF were not included in the study. CTD-ILD and IPF were two major ILD categories in our ILD cohort, while cases of disease such as hypersensitivity pneumonitis and sarcoidosis were not sufficient in number for comparison. In addition, we focused on the two groups (CTD-ILD and IPF), as they show different levels of autoimmune tendencies [15, 16]. Lastly, the treatment data in our study included only antifibrotics and short-term systemic steroids, and did not include detailed analysis of other treatment modalities such as lung transplant or immunosuppresives. In addition, baseline comorbidities of patients were not described. We think that a future study including more detailed treatment data and comorbidities is necessary.

## Conclusion

The IPAF group showed distinct clinical characteristics. In terms of ILD exacerbation and mortality, the IPAF group showed better clinical outcomes when compared to the IPF group. Further prospective studies are necessary to clarify the essential clinical features of IPAF before applying the disease category for clinical use.

## Data Availability

JUL and YHK are the guarantor of the content of this article, including the data and the analysis. All the data and the materials are available upon reasonable request.

## Abbreviations

AE: Acute exacerbation
ANOVA: One-way analysis of variance
Anti-CCP: Anti-cyclic citrullinated peptide
CTD-ILD: Connective tissue disease related-interstitial lung disease
DLCO: Diffusing capacity for carbon monoxide
FEV1/FVC: Forced expiratory volume in one second/forced vital capacity
HRCT: High resolution computed tomography
ILD: Interstitial lung disease
IPAF: Interstitial pneumonia with autoimmune features
IPF: Idiopathic pulmonary fibrosis
NSIP: Non-specific interstitial pneumonia
OS: Overall survival
PH: Pulmonary hypertension
RA: Rheumatoid arthritis
SLE: Systemic lupus erythematous
UCTD-ILD: Undifferentiated-CTD interstitial lung disease
UIP: Usual interstitial pneumonia
